# Predictive clinical factors for the progression from idiopathic late-onset cerebellar ataxia to multiple system atrophy cerebellar type

**DOI:** 10.1007/s00415-025-13292-w

**Published:** 2025-08-07

**Authors:** Seungmin Lee, Seoyeon Kim, Bora Jin, Su Hyeon Ha, Chanhee Jeong, Jung Hwan Shin, Han-Joon Kim

**Affiliations:** 1 Department of Neurology, Seoul National University Hospital, Seoul National University College of Medicine, 101 Daehak-ro, Jongno-gu, Seoul, 03080 Republic of Korea; 2 Department of Neurology, Inha University Hospital, Inha University College of Medicine, Incheon, Republic of Korea; 3 Department of Neurology, Dong-A University Hospital, Dong-A University College of Medicine, Busan, Republic of Korea

**Keywords:** ILOCA, MSA-C, NFL

## Abstract

**Objectives:**

Patients initially diagnosed with idiopathic late-onset cerebellar ataxia (ILOCA) may develop multiple system atrophy cerebellar type (MSA-C). However, data on conversion time, associated clinical factors, and the role of the neurofilament light chain (NFL) in this process remain limited. This study aims to investigate the conversion from ILOCA to MSA-C and examine the associated factors and the predictive value of NFL.

**Methods:**

This retrospective study included patients with ILOCA at the initial visit, recording the conversion to MSA-C and its duration. The median time to conversion was estimated using the Kaplan–Meier analysis. The roles of baseline orthostatic dizziness, rapid eye movement sleep behavior disorder (RBD), hot cross-bun sign, and urinary symptoms in conversion were analyzed. In a subset of patients, NFL levels in plasma collected before conversion were measured to examine their ability to predict conversion.

**Results:**

Seventy-two patients with ILOCA at the initial visit were included, of whom 32 experienced conversion to MSA-C. The median time-to-conversion was 5.0 years. RBD or orthostatic dizziness at baseline was associated with conversion, whereas hot cross-bun sign and urinary symptoms demonstrated no significant effect. Receiver operating characteristic analysis revealed moderate discriminative ability (area under the curve: 0.69) when NFL, orthostatic dizziness, and age at blood collection were included.

**Conclusion:**

This study identified that orthostatic dizziness and RBD, assessable in outpatient settings, correlated with ILOCA-to-MSA-C progression. Additionally, the NFL may play an auxiliary role in estimating the conversion time. Therefore, further studies incorporating diverse clinical and serological markers are required.

**Supplementary Information:**

The online version contains supplementary material available at 10.1007/s00415-025-13292-w.

## Introduction

Multiple system atrophy (MSA) is a rapidly progressive neurodegenerative disorder with core features, including cerebellar ataxia (CA), Parkinsonism, and autonomic dysfunction [1]. The initial clinical manifestation of MSA varies and may present as isolated CA, Parkinsonism, or autonomic failure, with a median survival of approximately 6–10 years [2, 3]. Patients require a wheelchair on average within 5 years [1, 4, 5].

Idiopathic late-onset CA (ILOCA) is a mixture of heterogeneous conditions, and some cases of idiopathic late-onset CA (ILOCA) eventually progress to the MSA-cerebellar type (MSA-C) [6], various other causes of CA should be considered. ILOCA, which does not progress to MSA-C, has been demonstrated to follow a lifelong disease trajectory and is thus considered a different disease entity [7]. Therefore, a thorough evaluation to differentiate between the hereditary, immunological, metabolic, inflammatory, and degenerative conditions that manifest as CA is essential for accurate diagnosis, prognostication, and therapeutic planning [5, 8].

Previous studies have reported that autonomic symptoms and rapid eye movement sleep behavior disorder (RBD) are more prevalent in MSA-C than in ILOCA. Additionally, cerebellar symptoms tend to be severe in patients with MSA-C [4, 9]. The hot cross-bun (HCB) sign is a radiological finding characterized by a cruciform T2 hyperintense signal on magnetic resonance imaging (MRI) of the pons. This is believed to reflect gliosis of the pontocerebellar fibers [10]. Although the presence of HCB on brain MRI within three years of symptom onset may help differentiate MSA-C from hereditary spinocerebellar ataxia (SCA) [11], limited reports have highlighted its utility in differentiating ILOCA from MSA. Moreover, studies on the clinical course of ILOCA and the conversion of the condition to MSA-C are lacking. Only a few studies have analyzed the time to conversion and investigated the clinical factors associated with MSA-C and ILOCA [7], especially using the 2022 Movement Disorder Society (MDS) MSA diagnostic criteria [12].

Neurofilament light chain (NFL) is a well-established biomarker for several neurodegenerative diseases, serving as an indicator of axonal damage or neuronal degeneration [13, 14]. Previous studies have demonstrated that plasma NFL is a reliable biomarker for assessing disease severity and monitoring MSA progression [15, 16]. NFL effectively differentiates MSA-C from healthy controls (HC); however, the ability of the chain to distinguish MSA-C from its mimic, SCA, may be limited compared to other biomarkers such as glial fibrillary acidic protein (GFAP) [17]. Furthermore, the ability of the NFL to differentiate ILOCA from prodromal MSA-C, to predict conversion from CA to MSA-C, has not yet been reported.

Therefore, this study aimed to investigate the proportion of patients with ILOCA who progressed to MSA-C, and to estimate the time to conversion. Additionally, clinical factors associated with conversion were examined, and the ability of plasma NFL to differentiate between the two groups was evaluated.

## Methods

### Study participants

The medical records of patients with ILOCA who visited the Movement Disorder Clinic at Seoul National University Hospital (SNUH) between January 2019 and February 2025 were retrospectively reviewed. Patients were diagnosed with ILOCA based on the following inclusion criteria: (i) progressive CA with symptom onset after the age of 30 years; (ii) no family history of ataxia; (iii) no laboratory findings suggestive of non-degenerative causes such as inflammatory, metabolic or autoimmune conditions; and (iv) absence of genetic findings indicative of hereditary ataxias. The 2022 MDS criteria were applied to diagnose MSA [12] and patients who had already met the criteria for probable or established MSA at the time of the study were excluded. The study protocol was approved by the Institutional Review Board (IRB) of Seoul National University Hospital (IRB No. 2406-073-1544). The study was conducted following the principles of the Declaration of Helsinki.

### Clinical and laboratory data collection

Demographic and clinical data, as well as laboratory test results, were obtained by reviewing medical records. The conversion time from ILOCA to MSA-C was defined as the date when a patient's medical record met the 2022 MDS criteria for probable or established MSA-C, marking the patient as an MSA-C converter. The Hoehn and Yahr (H&Y) scale was employed at the time of conversion to MSA-C. The unified MSA rating scale (UMSARS), along with the Mini-Mental State Examination (MMSE) and Montreal Cognitive Assessment (MoCA) results from the initial visit, were collected from medical records. Data on urinary frequency and urgency were gathered from medical records.

Additionally, the presence of orthostatic dizziness and probable RBD was assessed, with RBD diagnosed using the Rapid Eye Movement Sleep Behavior Disorder Questionnaire (RBDIQ) [18] or polysomnography (PSG). Thirty-two patients were diagnosed with RBD1Q, and five patients were diagnosed based on PSG. The HCB sign was assessed on T2-weighted axial images at the pontine level and was considered present only if vertical or both horizontal and vertical lines were observed.

To exclude non-degenerative causes of CA, results of cerebrospinal fluid (CSF) studies, CSF panels for autoimmune encephalitis, serum paraneoplastic antibodies, genetic testing for SCA types 1, 2, 3, 6, 7, 8, and 17, as well as dentatorubral-pallidoluysian atrophy (DRPLA), next generation sequencing (NGS), anti–glutamic acid decarboxylase antibodies, thyroid function tests, vitamin B12, folate, and vitamin E levels were reviewed. Detailed description about laboratory tests is listed in the Supplementary Table 1.

### Plasma collection and measurement of NFL

Plasma concentration of NFL was assessed in 42 participants whose blood samples were provided and stored at − 80 °C. The concentration of plasma NFL was measured using ultrasensitive single-molecule array (Simoa®) technology (Quanterix, Billerica, MA, USA) on the automated Simoa HD-X platform. The mean of the duplicate values was used as the outcome. Stored plasma obtained from age- and sex-matched healthy participants was used as the control.

### Statistical analysis

The normality of continuous variables was assessed using the Shapiro–Wilk test. Levene’s test was used to evaluate the homogeneity of variance. For comparison between the MSA-C converter and non-converter, continuous variables were assessed using either the *t*-test or Wilcoxon rank-sum test, while categorical variables were analyzed using the chi-square or Fisher’s exact tests. Three-group comparisons were performed using analysis of variance with Tukey's test for post-hoc analysis or the Kruskal–Wallis test with Dunn's test, as appropriate. Time-to-event analysis was performed using the Kaplan–Meier curve with the *survival* and *survminer* packages in R (version 3.7-0 and 0.4.9, respectively). The log-rank test was used to compare the transition times between groups. The relationships between two variables were evaluated using Pearson’s or Spearman’s correlation analyses, depending on the distribution of the data. Receiver operating characteristic (ROC) curve analysis was used to assess the ability of NFL or clinical variables to differentiate converters from non-converters, with diagnostic performance expressed as the area under the curve (AUC). Statistical significance was defined as a two-tailed P value less than 0.05. All statistical analyses were conducted using R version 4.3.3 (http://www.r-project.org).

## Results

### Demographics and characteristics of study participants

A total of 323 patients with CA visited over a period of 6 years and 2 months. Among them, 142 who fulfilled the criteria for probable or possible MSA at their initial visit were excluded. Additionally, 67 patients diagnosed with hereditary CA, 16 with autoimmune or inflammatory causes, eight younger than 30 years, and 18 with a family history of ataxia were excluded. Consequently, 72 patients with ILOCA were included in this study, with 30 experiencing conversion to MSA-C during the observation period (Supplementary Fig. 1). Lumbar puncture, CSF AE panel, and NGS were performed only in a subset of patients (CSF study and AE panel: 33 patients, 45.8%; NGS: 4 patients, 5.6%). None of the patients with ILOCA showed abnormal findings based on our predefined criteria.

No significant differences were observed in age of onset, sex, or disease duration between the converter and non-converter groups. Twenty-two patients (52.4%) in the non-converter group were lost to follow-up during the study period. RBD was more prevalent in the MSA converter group, with a trend toward statistical significance (*p* = 0.051). Urinary symptoms, orthostatic dizziness, and prevalence of the HCB sign did not differ between the groups. Additionally, no statistical difference was noted in the two cognitive scores or the UMSARS scores between the groups (Table 1). In the converter group, most patients were classified as having H&Y stage 4 or 5 (stage 3, *n* = 5; stage 4, *n* = 21; stage 5, *n* = 4). No death was reported during the study period.

**Table 1:** The demographics, clinical parameters, and study results of the overall group at the initial visit

	MSA-C converter (*n* = 30)	Non-converter (*n* = 42)	*p*-value
Age of onset (year)	56.9 ± 7.3	55.0 ± 9.3	0.60
Disease duration (year)	1.7 ± 1.5	1.9 ± 1.4	0.27
Onset to convert (year)	3.7 ± 1.6	-	
Onset to last visit (year)	-	3.7 ± 2.2	
Male: Female	17:13	17:25	0.26
RBD	20 (66.7)	17 (40.5)	0.051
urinary frequency	15 (50.0)	14 (33.3)	0.24
urinary urgency	11 (36.7)	7 (16.7)	0.10
orthostatic dizziness	5 (16.7)	4 (9.5)	0.48
HCB sign	13 (43.3)	11 (26.2)	0.20
UMSARS part I	8.5 ± 3.6 (*n* = 10)	8.9 ± 5.0 (*n* = 25)	0.81
UMSARS part II	12.5 ± 6.5 (*n* = 10)	11.0 ± 5.9 (*n* = 25)	0.54
MMSE	27.1 ± 1.2 (*n* = 13)	26.8 ± 2.7 (*n* = 21)	0.77
MoCA	24.7 ± 2.3 (*n* = 13)	24.1 ± 4.4 (*n* = 17)	0.61

Data are presented with number (percent), or mean ± standard deviation

*HC* healthy control, *HCB* Hot cross-bun, *MMSE* Mini-Mental Status Examination, *MoCA* Montreal Cognitive Assessment, *MSA-C* Multiple system atrophy cerebellar type, *RBD* rapid eye movement sleep behavior disorder, *UMSARS* Unified multiple system atrophy rating scale

Samples for plasma NFL analysis were available from 21 non-converter patients and 21 converters (NFL group, *n* = 42). Twenty-six age- and sex-matched controls were analyzed. Given that plasma samples were obtained at various time points, age and UMSARS scores at the time of sampling were analyzed separately in each patient subset. No significant differences in clinical parameters between non-converters and converters in the NFL group were observed (Supplementary Table 2).

### Time-to-event analysis for the transition from ILOCA to MSA-C

**Fig. 1:**
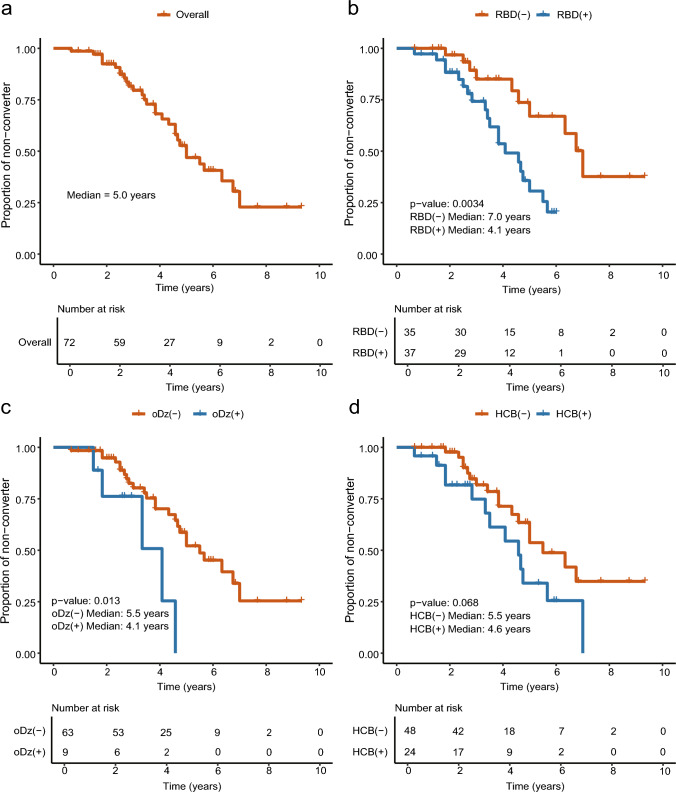
Kaplan–Meier curves for progression to MSA-C in non-converters and converters. Kaplan–Meier curve for **a** Overall group, **b** Stratified by the RBD, **c** Stratified by the oDz, **d** Stratified by the HCB. + denotes censored data points. *HCB* Hot cross-bun; *ILOCA* Idiopathic late-onset cerebellar ataxia; *MSA-C* Multiple system atrophy cerebellar type; *oDz* orthostatic dizziness; *RBD*, rapid eye movement sleep behavior disorder

In the overall group, the median time to conversion to MSA-C was 5.0 years (Fig. 1a). Patients who reported documented RBD or orthostatic dizziness had significantly reduced conversion times than those who did not (*p* = 0.0034 for RBD and *p* = 0.013 for orthostatic dizziness) (Fig. 1b, c). The HCB sign on MRI was associated with a short time to MSA-C diagnosis; however, the difference was not statistically significant (*p* = 0.068) (Fig. 1d). Sex was not a determining factor (median time (year): men 5.5; women, 4.6; *p* = 0.80). Urinary symptoms did not impact conversion time (Supplementary Fig. 2a, b). In the NFL group, the median time to conversion was 4.6 years. The presence of RBD was significantly associated with rapid conversion (*p* = 0.0071), whereas orthostatic dizziness and HCB sign revealed no significant association (Supplementary Fig. 3a–d).

### Evaluation for the NFL as a potential predictive marker for conversion

**Fig. 2:**
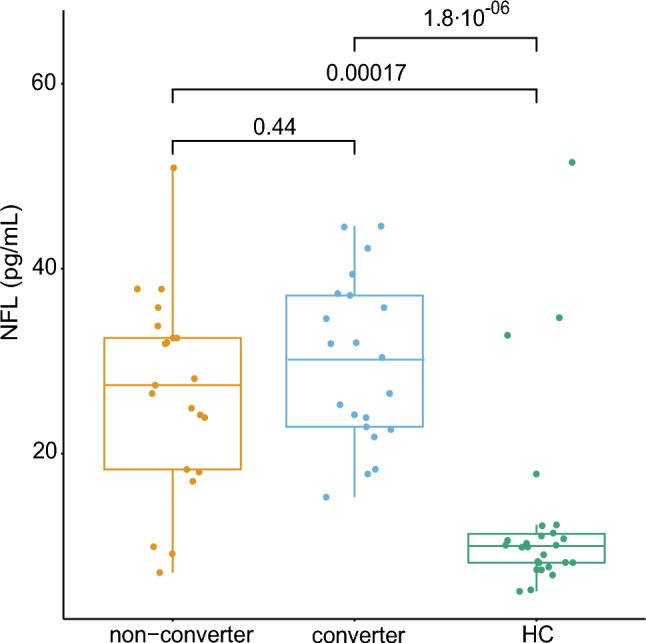
Boxplots illustrating plasma NFL concentrations among the three groups. *HC* healthy control, *NFL* neurofilament light chain

The two patient groups exhibited significantly elevated plasma NFL levels compared with those observed in the HC group, whereas no difference between the two groups was noted (29.9 ± 9.0 pg/mL for converter, 26.6 ± 10.8 pg/mL for non-converter, 13.0 ± 10.6 pg/mL for HC) (Fig. 2). Dichotomization of the patients based on six clinical variables (sex, RBD, orthostatic dizziness, HCB, urinary frequency, and urinary urgency) revealed no significant differences (Supplementary Fig. 4a–f). Age at sampling and NFL levels were significantly correlated only in the HC group. No correlation was observed between NFL and disease duration at the time of sampling. NFL levels exhibited a trend toward negative correlation with time to conversion (*r* = −0.42, *p* = 0.065) (Supplementary Fig. 5f). UMSARS demonstrated no correlation with non-converters or converters (Supplementary Fig. 6a–d).

**Fig. 3:**
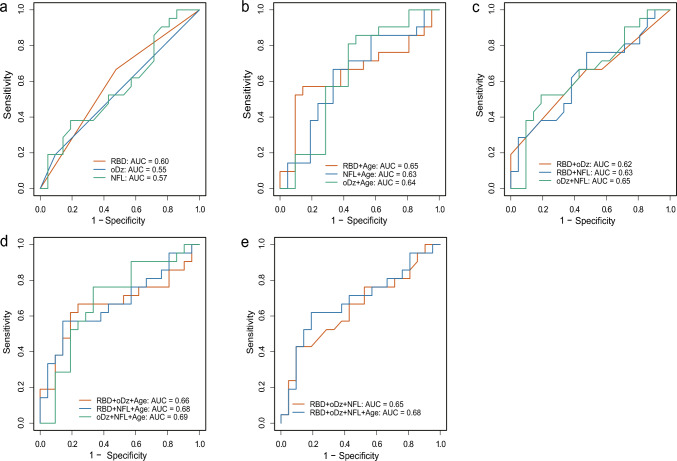
ROC curves from binomial logistic regression models evaluating the discriminative performance of plasma NFL levels and clinical variables, either alone or in combination, for predicting conversion to MSA-C. **a** Models using each variable individually (RBD, oDz, and NFL). **b** Two-variable models combine each individual variable with age at sampling. **c** Two-variable models using combinations of clinical parameters. **D** Three-variable models, including age at sampling added to those in (**c**). **e** Models incorporating three or more variables. Age refers to age at the time of sample collection. *AUC* Area under the curve, *MSA-C* Multiple system atrophy cerebellar type, *oDz* Orthostatic dizziness, *NFL* Neurofilament light chain, *RBD* Rapid eye movement sleep behavior disorder, *ROC* Receiver operating characteristic

In the ROC analysis, NFL alone demonstrated limited discriminative performance (AUC = 0.57, 95% confidence interval [CI] 0.39–0.75). RBD, orthostatic dizziness, all significant in the log-rank test, and age at sampling, were subsequently included in the ROC analysis. Models incorporating any combination of the two variables were better than those with any single variable. The inclusion of age at sampling further improved model accuracy. The optimum performance was observed in the model combining orthostatic dizziness, NFL, and age at sampling (AUC = 0.69, 95% CI 0.50–0.85) (Fig. 3a–e).

In the NFL group, 11 non-converters were followed up for less than 3 years from symptom onset, raising the possibility that some may have eventually progressed to MSA-C but were not diagnosed due to the short follow-up period. Among the 11 patients, RBD was diagnosed in eight, orthostatic dizziness in two, and HCB sign in six. In contrast, among 10 non-converters who were followed up for more than 3 years, only two patients demonstrated the HCB sign. Moreover, RBD was noted in two individuals, meanwhile, orthostatic dizziness was not reported at the initial visit. The 3-year cutoff was selected based on the 2022 MDS MSA diagnostic criteria, which defines fast progression as progression within 3 years [12]. When the analysis was repeated, including only the 10 non-converters who had been followed up for at least 3 years, the level of NFL still demonstrated no significant difference between converters and non-converters (Supplementary Fig. 7). However, the overall AUC increased, with the model including RBD, orthostatic dizziness, NFL, and age at sampling having the highest AUC at 0.80 (Supplementary Fig. 8).

## Discussion

In the present study, the median time from ILOCA to MSA‐C conversion was 5 years. RBD or orthostatic dizziness was associated with a significantly reduced conversion time. Although NFL levels alone did not correlate with either disease severity or discrimination between the two groups, the addition of NFL improved the discriminative ability of RBD or orthostatic dizziness, reaching an AUC of 0.69 when orthostatic dizziness, NFL levels, and age at sampling were considered together.

A previous study reported that 24% of patients with sporadic olivopontocerebellar atrophy (sOPCA) progress to MSA within 5 years of symptom onset [7]. Another study identified that disease progression in MSA, particularly dependence on walking aids, is significantly faster than that in unexplained sporadic ataxia [9]. In our study, 23 (42.6%) converters were identified within 5 years. However, a direct comparison with the outcomes of the previous studies is challenging, as they adopted the MSA diagnostic criteria of Gilman et al. [19]. Furthermore, genetic studies were performed only for SCA1, 2, and 3 [7, 9]. Conversely, our patients underwent an extensive genetic workup and evaluation for diverse etiologies, excluding patients with other etiologies.

Although the baseline proportion of patients with RBD did not differ significantly between converters and non-converters, those with RBD exhibited a significantly short median conversion time. In a previous study, RBD was not observed at the initial or cumulative visits in the ILOCA group, where the condition was diagnosed based on family-reported abnormal motor behaviors during sleep [4]. In contrast, our study identified RBD in some patients within the non-converter group. Multiple factors may have contributed to this outcome. Although the RBD1Q utilized in this study had a high specificity and sensitivity [18], the possibility of false positives cannot be ruled out [20]. In addition, some patients with RBD may have dropped out before conversion, potentially affecting the observed conversion rates. Another explanation is that RBD in non-converters may not necessarily be a prodrome of MSA, given that RBD is not exclusive to synucleinopathies and can also occur in brainstem lesions and cerebellar disorders such as SCA [21, 22]. However, since patients with RBD preceding the onset of MSA have been reported to exhibit a poor prognosis and rapid progression [23, 24], the association between a short median time and the presence of RBD in our study appears reasonable.

The prevalence of the HCB sign did not differ between the two groups, nor was the sign associated with a short median time. As the HCB sign is not pathognomonic for MSA and has been observed in a range of other neurological disorders with cerebellar degeneration [25, 26], the presence of HCB in non-converters is not unusual. However, considering the relatively short follow-up period in this study, whether non-converters with the HCB sign will eventually evolve into MSA-C remains unclear. Vertical or cruciform hyperintensity in the pons in the early stage of disease has been reported to be highly specific for MSA, the prevalence ranging from 45 to 90% in patients with MSA-C, whereas being seen in less than 1% of patients with spinocerebellar ataxias [10, 11, 27]. Thus, further follow-up studies are required to elucidate the significance of these findings in ILOCA.

Conflicting findings exist in the literature regarding the correlation between plasma NFL levels and the UMSARS. Although some studies have reported a significant association [15, 16], others have not identified a direct correlation [28]. In our study, no significant association was observed between the plasma NFL level and UMSARS scores. However, an increasing trend in plasma NFL levels was observed as the patients approached the time of MSA-C diagnosis. Considering previous findings that plasma NFL levels remain stable over time in early MSA-C despite worsening clinical scores [29], our results suggest that NFL levels may plateau around the time of MSA-C diagnosis. This discrepancy may also be attributed to the relatively small sample size of our study.

NFL levels did not demonstrate robust performance in discriminating converters from non-converters. The discriminative ability improved when NFL levels were combined with clinical variables. These findings highlight the limited utility of the NFL as a standalone biomarker, possibly reflecting its lower disease specificity [30], while also suggesting that incorporating additional contributing factors may enhance the overall model performance. The improved model performance observed in individuals with follow-up longer than 3 years suggests that the predictive ability of NFL may be greater than that in our study for patients with CA with a prolonged disease duration.

Our study had certain limitations. First, the high dropout rate in the non-converter group may have influenced the results of the time-to-event analysis. As MSA-C features, such as orthostatic dizziness and RBD, were also observed in the non-converter group, a prolonged follow-up period might have revealed additional cases of conversion. Secondly, the diagnosis of MSA was not confirmed pathologically. However, we attempted to minimize this risk by performing genetic workups and additional laboratory tests to exclude potential genetic, autoimmune, and inflammatory causes. Third, only five diagnoses were confirmed by PSG, which may have influenced the results; however, the prevalence of RBD in the converter group was consistent with the outcomes of the previous studies [31]. Fourth, our study screened for genetic ataxias, such as SCA1, 2, 3, 6, 7, 8, and 17, and DRPLA, primarily focusing on those associated with trinucleotide repeat expansions. NGS was performed in only four patients, and thus, we could not completely exclude rare hereditary ataxias. In particular, testing for SCA27b and Cerebellar Ataxia, Neuropathy, and Vestibular Areflexia Syndrome (CANVAS) was not available. Although no patients exhibited episodic ataxia, one case with polyneuropathy and slowly progressive ataxia was excluded, but the possibility of these two conditions remains. To minimize this possibility, we excluded patients with a family history of ataxia, even if their genetic testing was negative. Fifth, although we performed paraneoplastic and autoimmune antibody panels, testing for anti- Homer-3 antibody and anti-ZIC4, which could be possible cause of CA [32], was not available at our institution, and thus these possibilities could not be completely ruled out. In addition, CSF studies were not performed in all participants. Finally, considering not all variables were assessed longitudinally, we were unable to present the changes in these variables according to disease progression. In particular, although baseline UMSARS scores were recorded for some patients, longitudinal assessments were not available, and thus UMSARS was not included in the analysis of disease progression. Further longitudinal studies are required to evaluate these aspects.

In conclusion, our study estimated the time to conversion to MSA-C and the clinical factors associated with a fast conversion, with most recent MDS MSA diagnostic criteria [12]. Moreover, the assessment of RBD and orthostatic dizziness in routine clinical practice may assist in predicting the progression of ILOCA. Although the NFL as a single marker demonstrated limited predictability, its combination with other clinical variables helped differentiate between non-converters and converters. α-Synuclein seed amplification assays such as RT-QuIC and protein misfolding cyclic amplification have been studied for their ability to differentiate MSA from Parkinson’s disease and to distinguish between MSA subtypes [33, 34], and applying these assays to patients with ILOCA may be worthwhile to determine whether some are in a prodromal stage of MSA rather than having truly idiopathic ataxia. This study also suggested a possible association between NFL levels and the time to MSA-C conversion, indicating its potential role in reflecting disease progression rate and predicting disease progression. These findings highlight the need for further studies incorporating a broad range of biomarkers to improve the prediction of conversion, which is crucial for determining optimal treatment strategies and prognostication.

## Supplementary Information

Below is the link to the electronic supplementary material.

Supplementary file1 (PDF 859 KB)

## Data Availability

The data used in the current study are available from the corresponding author on reasonable request.
