# Supplementary material for: Predictive clinical factors for the progression from idiopathic late-onset cerebellar ataxia to multiple system atrophy cerebellar type
Source: J Neurol. 2025 Aug 7;272(9):561. doi: 10.1007/s00415-025-13292-w (PMC12331855; doi:10.1007/s00415-025-13292-w)
Supplement: Supplementary file 1 — Supplementary file1 (PDF 859 KB) [file 415_2025_13292_MOESM1_ESM.pdf]

Predictive clinical factors for the progression from idiopathic late-onset cerebellar ataxia to multiple system atrophy cerebellar type

Seungmin Lee, MD[1], Seoyeon Kim, MD[1], Bora Jin, MD[1], Su Hyeon Ha, MD[1], Chanhee Jeong, MD[1], Jung Hwan Shin, MD, PhD[1], Han-Joon Kim, MD, PhD [1*]

[1]Department of Neurology, Seoul National University Hospital, Seoul National University College of Medicine, Seoul, Republic of Korea

Corresponding author: Han-Joon Kim, MD, PhD

Corresponding author's address: Department of Neurology, Seoul National University College of Medicine, 101 Daehak-ro, Jongno-gu, Seoul 03080, South Korea

Corresponding author's phone and fax: +82-2-2072-2278 and +82-2-3672-7553

Corresponding author's e-mail address: movement@snu.ac.kr

Supplementary Table 1. Detailed description of genetic, metabolic, paraneoplastic, autoimmune, and cerebrospinal fluid (CSF) investigations performed in this study

**Genetic test**
Spinocerebellar ataxia (SCA) 1,2,3,6,7,8,17, dentatorubro-pallidoluysian atrophy (DRPLA)<br>Ataxia gene panel: ABCD1, APTX, ATL1, ATM, BEAN1, CACNA1A, CACNB4, CP, CYP27A1, FMR1, FXN, GFAP, KCNA1, MTTP, NPC1, PNKP, POLG, SACS, SETX, SLC1A3, SPAST, SPG11, SPG7, SYNE1, TK2, TTPA, ZFYVE26, PLA2G6, ATP1A3, ANO10, RFC1, C10orf2, PHYH, PEX7, PNPLA6, SIL1, SLC52A2, SNX14, WFS1, CAPN1, COQ2
**CSF autoimmune encephalitis (AE) antibody study**
Alpha-amino-3-hydroxy-5-methyl-4-isoxazolepropionic acid receptor (AMPAR) antibody,<br>Contactin-associated protein-like 2 (CASPR2) antibody,<br>Dipeptidyl-peptidase-like protein 6 (DPPX) antibody,<br>Gamma-aminobutyric acid type B receptor ($GABA_BR$) antibody,<br>Leucine-rich glioma-inactivated 1 (LGI1) antibody,<br>N-methyl-D-aspartate receptor (NMDAR) antibody
**Metabolic**
Thyroid function test (thyroid stimulating hormone, Free T4, T3), vitamin B12, folate, Vitamin E
**Serum paraneoplastic antibody panel**
Amphiphysin, CV2 (CRMP5), PNMA2 (Ma2/Ta), Ri, Yo, Hu, Recoverin, SOX1, Titin (MGT-30)
**Other**
Serum Anti-glutamic acid decarboxylase (GAD) antibody

Supplementary Table 2. The demographics, clinical parameters and study results of NFL group

	MSA-C converter (n=21)	Non-converter (n=21)	HC (n=26)	p-value
Age of onset (year)	56.6 ± 5.6	54.1 ± 6.3	-	0.19
Age of sample (year)	58.5 ± 2.1	56.4 ± 6.2	56.6 ± 3.2	0.34
Disease duration (year)	1.8 ± 1.4	2.0 ± 1.4	-	0.74
Onset to convert (year)	3.6 ± 1.3	-	-	-
Onset to last visit (year)	-	3.6 ± 2.1	-	-
Male : Female	13:8	12:9	11:15	0.37
RBD	14 (66.7)	10 (47.6)	-	0.35
urinary frequency	8 (38.1)	8 (38.1)	-	1.0
urinary urgency	9 (42.9)	4 (19.0)	-	0.18
orthostatic dizziness	4 (19.0)	2 (9.5)	-	0.66
HCB sign	9 (42.9)	8 (38.1)	-	1.0
UMSARS part I	10.2 ± 5.4 (n=6)	11.3 ± 4.4 (n=14)	-	0.67
UMSARS part II	14.0 ± 7.0 (n=6)	14.8 ± 6.8 (n=14)	-	0.83
MMSE	27.2 ± 1.3 (n=10)	26.8 ± 3.4 (n=12)	-	0.46
MoCA	25.1 ± 2.4 (n=10)	24 ± 5.8 (n=9)	-	0.61

Data are presented with number (percent), or mean ± standard deviation

Abbreviations: HC, healthy control; HCB, Hot crossbun; MMSE, Mini-Mental Status Examination; MoCA, Montreal Cognitive Assessment; MSA-C, Multiple system atrophy cerebellar type; REM sleep behavior disorder, UMSARS, Unified multiple system atrophy rating scale

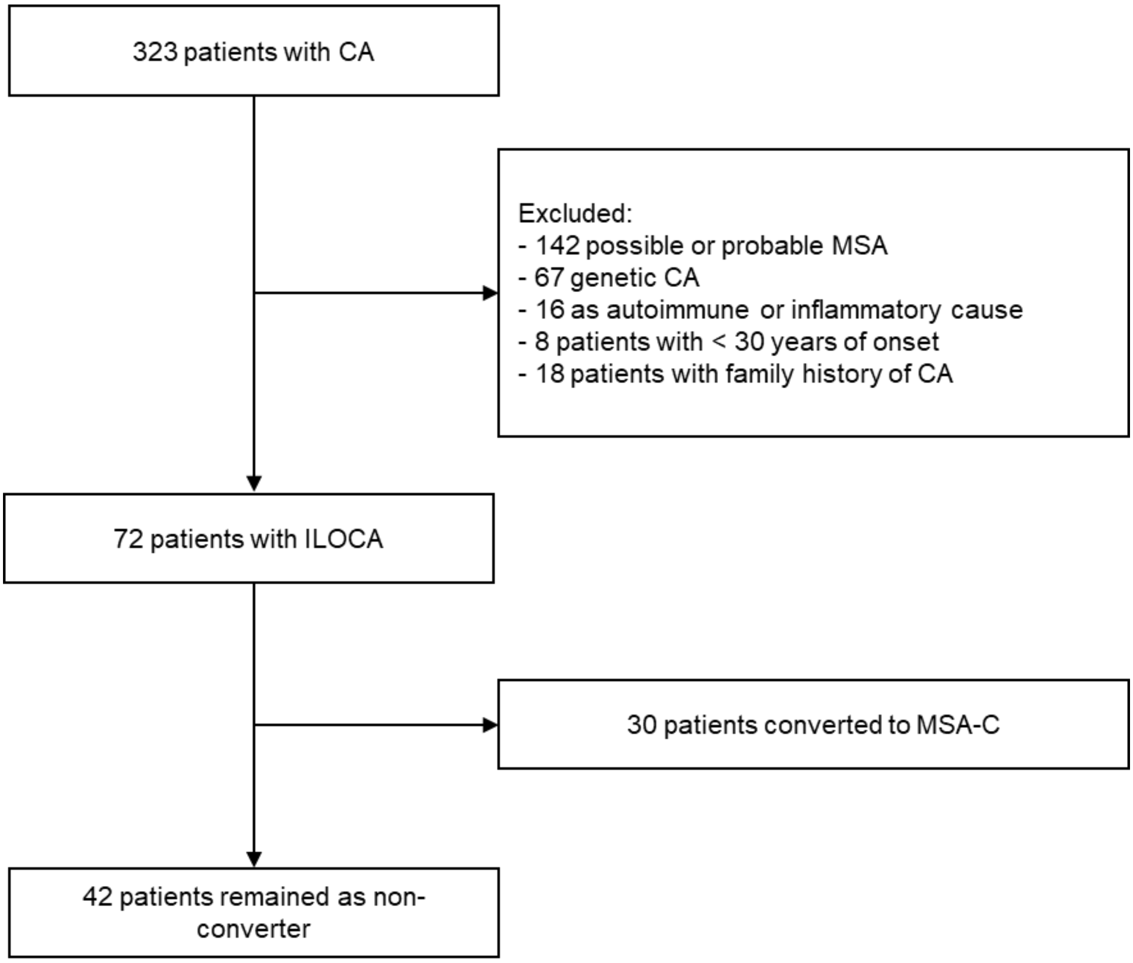

Supplementary Figure 1. Participant selection and classification flowchart according to inclusion and exclusion criteria

Abbreviations: CA, cerebellar ataxia; HCB, Hot cross-bun; ILOCA, Idiopathic late-onset cerebellar ataxia; MSA-C, Multiple system atrophy cerebellar type

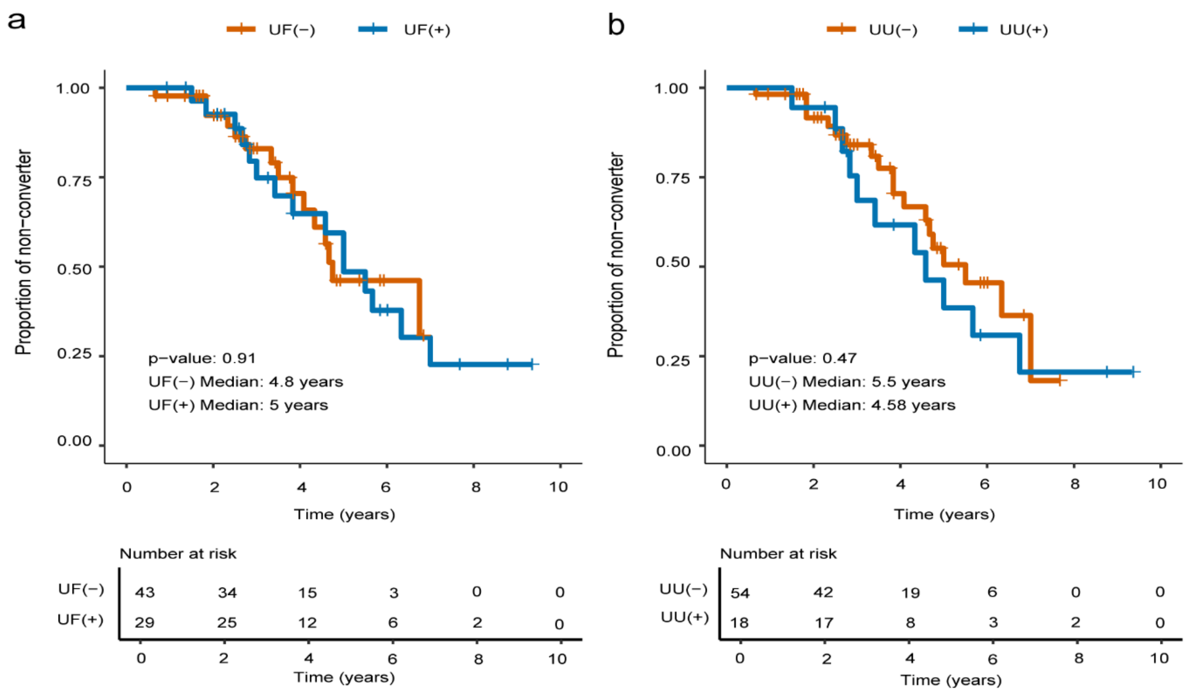

Supplementary Figure 2. Kaplan–Meier curves for progression to MSA-C in converters and non-converters stratified by urinary symptoms. Kaplan–Meier curve (**a**) stratified by urinary frequency (UF), (**b**) stratified by urinary urgency (UU). + denotes censored data points.

Abbreviations: ILOCA, Idiopathic late-onset cerebellar ataxia; MSA-C, Multiple system atrophy cerebellar type; UF, urinary frequency; UU, urinary urgency

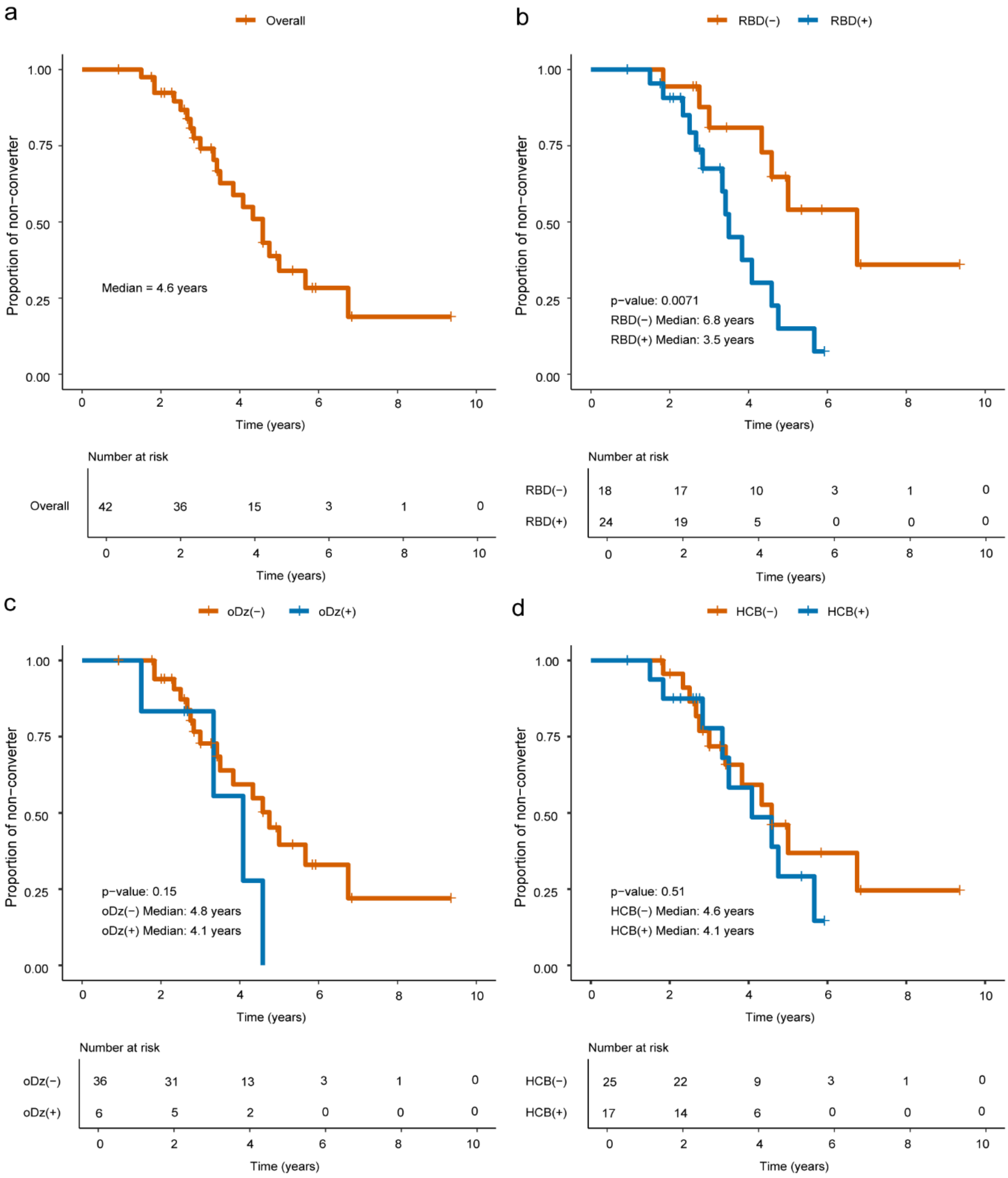

Supplementary Figure 3. Kaplan–Meier curves for conversion to MSA-C in patients in the NFL group. Kaplan–Meier curve for (**a**) NFL group, (**b**) stratified by the RBD, (**c**) stratified by the orthostatic dizziness (oDz), (**d**) stratified by the HCB. + denotes censored data points. Abbreviations: HCB, Hot cross-bun; MSA-C, Multiple system atrophy cerebellar type; oDz, orthostatic dizziness; RBD, REM sleep behavior disorder

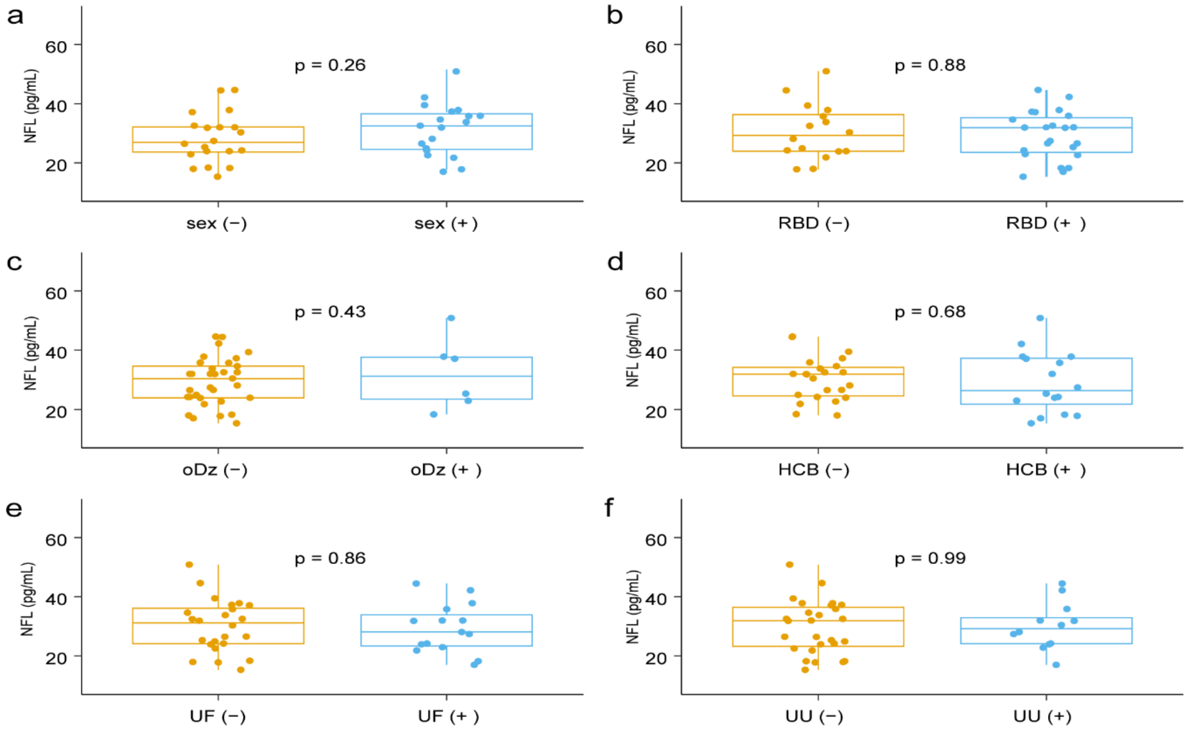

Supplementary Figure 4. Comparison of serum NFL levels according to sex (**a**) and the presence of RBD (**b**), HCB (**c**), orthostatic dizziness (oDz) (**d**), urinary frequency (UF) (**e**), and (**f**) urinary urgency. Differences between the two groups were assessed using the Wilcoxon rank-sum test. (N_{Male}: 25, N_{Female}: 17, $N_{RBD(+)}$: 24, $N_{RBD(-)}$:18, $N_{HCB(+)}$: 17, $N_{HCB(-)}$: 35 $N_{oDz(+)}$:6, $N_{oDz(-)}$:36, $N_{UF(+)}$:16, $N_{UF(-)}$:26, $N_{UU(+)}$:13, $N_{UU(-)}$:29)

Abbreviations: HCB, Hot cross-bun; ILOCA, Idiopathic late-onset cerebellar ataxia; MSA-C, Multiple system atrophy cerebellar type; oDz, orthostatic dizziness; RBD, REM sleep behavior disorder; NFL, neurofilament light chain; UF, urinary frequency; UU, urinary urgency.

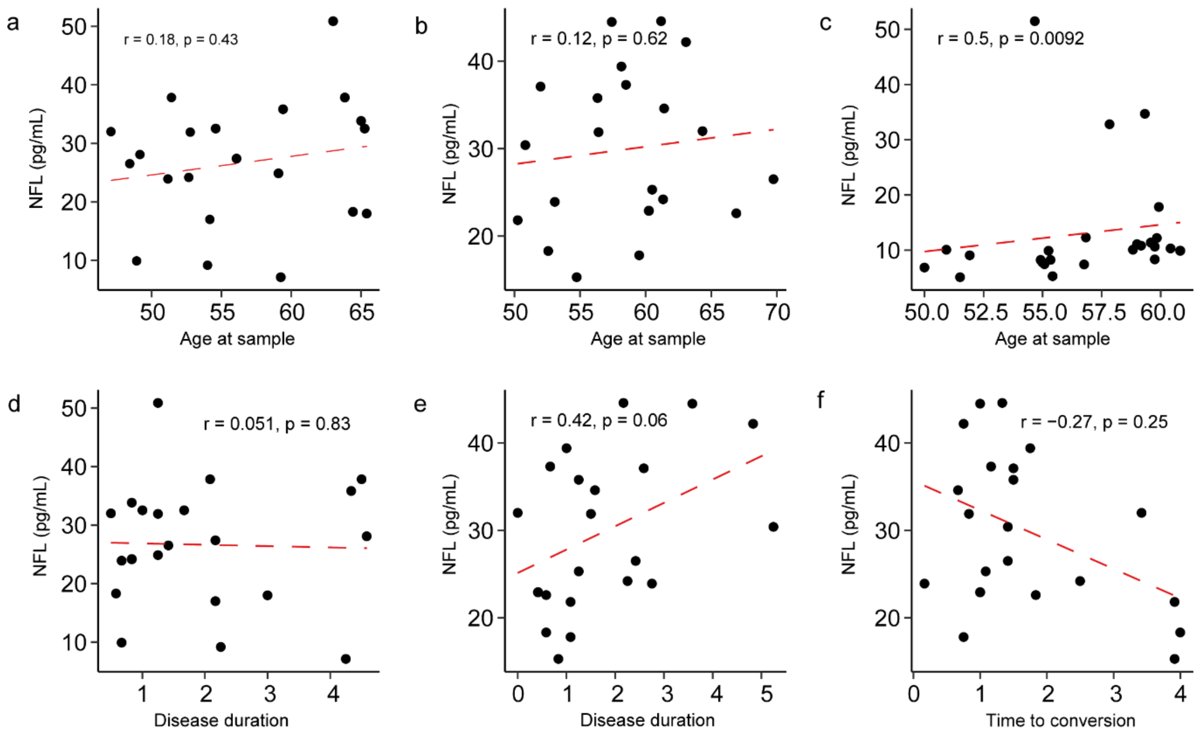

Supplementary Figure 5. The scatter plot displays the correlation between NFL and clinical parameters. Correlation between NFL and age at sample in (**a**) ILOCA group, (**b**) MSA-C converter group, (**c**) HC. Correlation between NFL and disease duration in (**d**) ILOCA group, (**e**) MSA-C converter group. (**f**) Correlation between NFL and time to conversion in the MSA-C converter group, using age at sample as covariates. ($N_{non\text{-}converter}$: 21, $N_{converter}$: 21, N_{HC}: 26)

Abbreviations: HCB, Hot cross-bun; ILOCA, Idiopathic late-onset cerebellar ataxia; MSA-C, Multiple system atrophy cerebellar type; OH, orthostatic hypotension; RBD, REM sleep behavior disorder

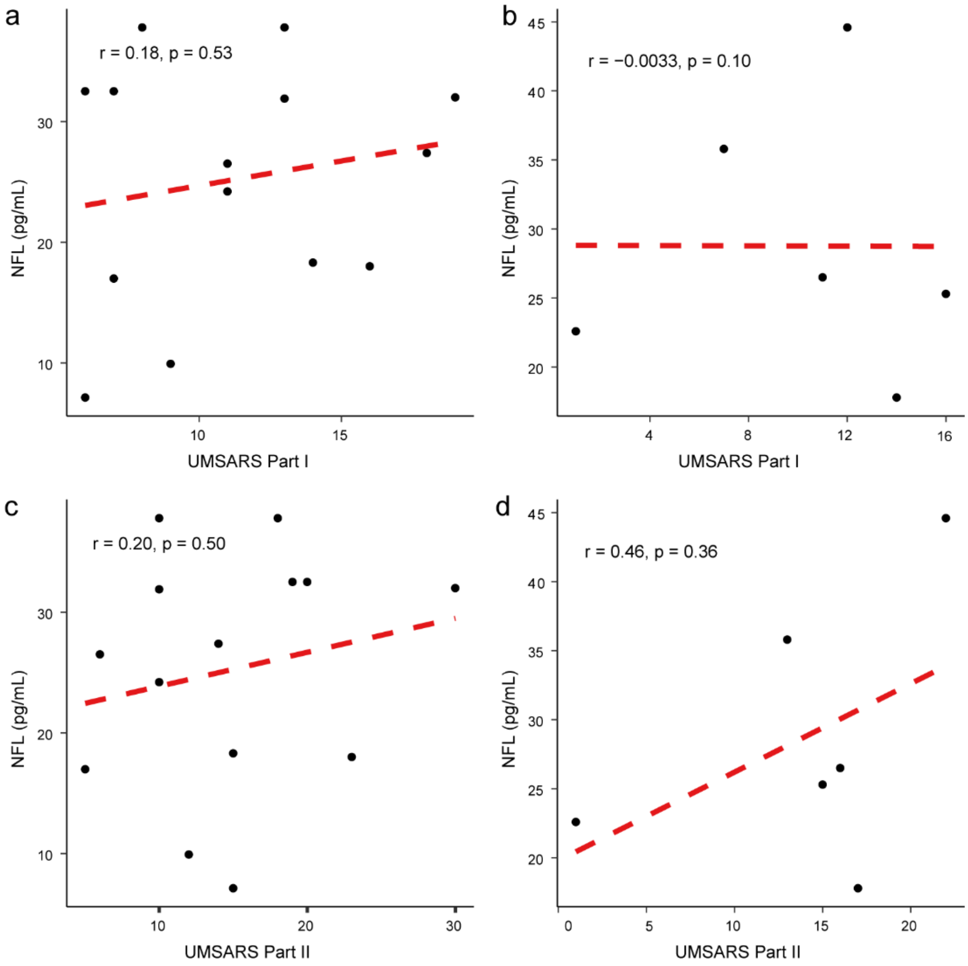

Supplementary Figure 6. The scatter plot illustrates the correlation between NFL and UMSARS. Correlation between NFL and UMSARS part I in (**a**) non-converter group, (**b**) converter group. Correlation between NFL and UMSARS part II in (**c**) non-converter group and (**d**) converter group. All correlations shown above were assessed using Spearman's correlation. ($N_{\text{UMSARS converter}}$ = 6, $N_{\text{UMSARS non-converter}}$ = 14)

Abbreviations: ILOCA, Idiopathic late-onset cerebellar ataxia; MSA-C, Multiple system atrophy cerebellar type; NFL, neurofilament light chain; UMSARS, unified multiple system atrophy rating scale

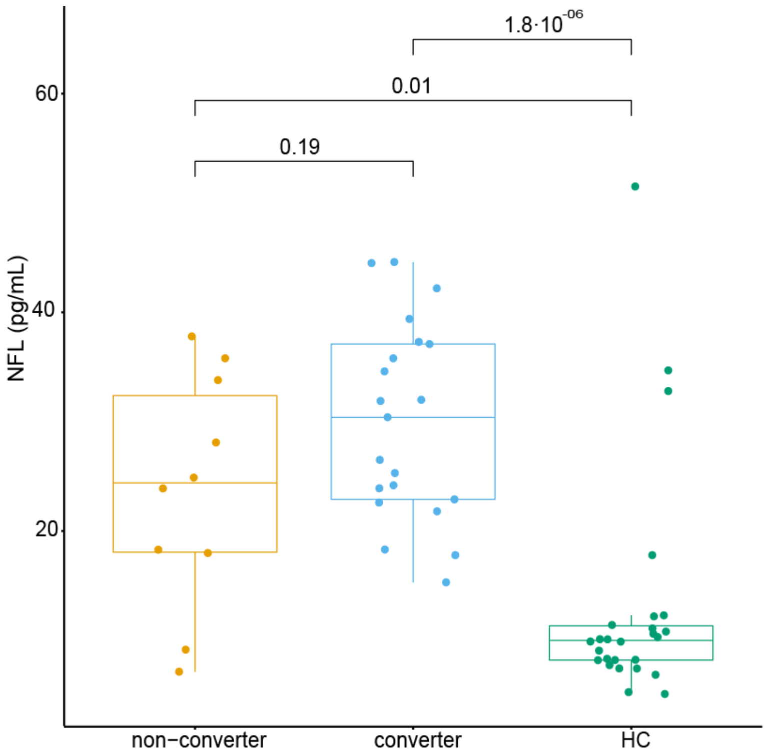

Supplementary Figure 7. Boxplots illustrating NFL concentrations across the three groups, including non-converters, followed for more than 3 years. ($N_{non\text{-}converter}$: 10, $N_{converter}$: 21, N_{HC}: 26)

Abbreviations: HC, healthy control; NFL, neurofilament light chain

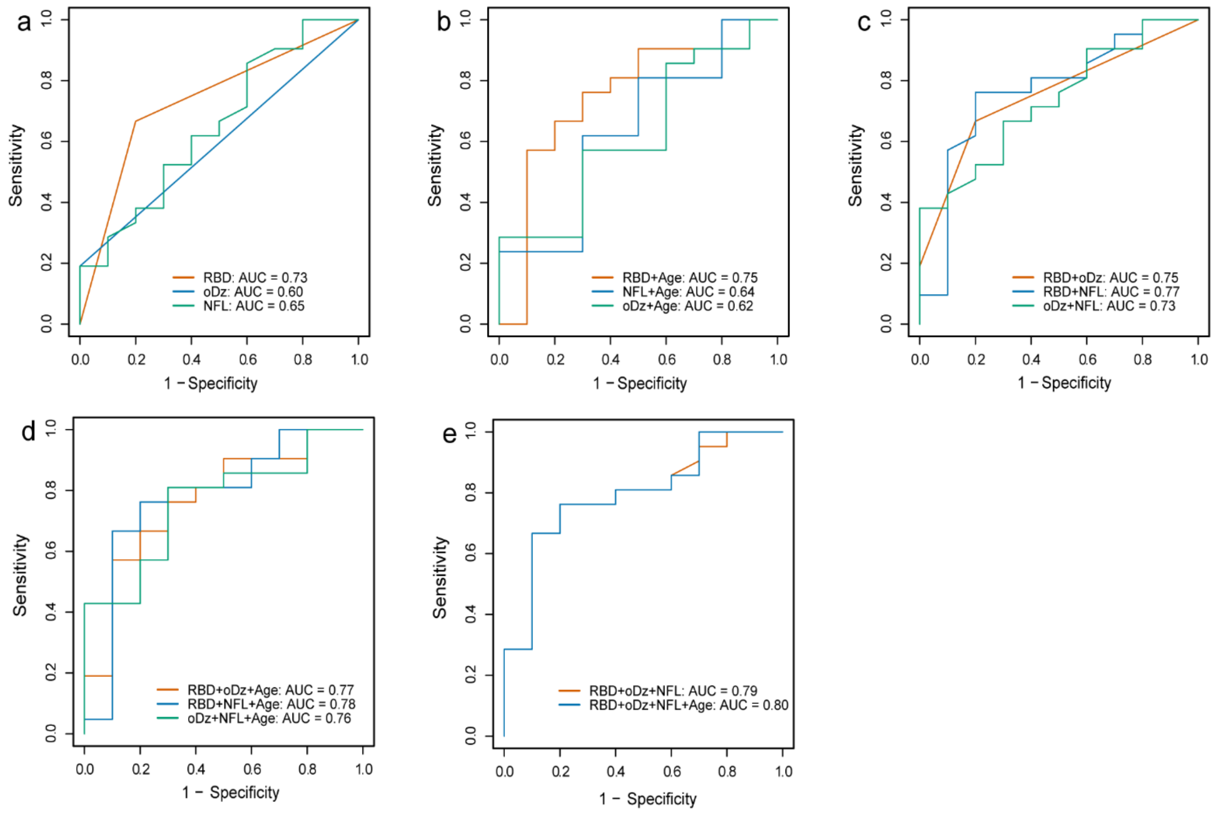

Supplementary Figure 8. ROC curves from binomial logistic regression models evaluating the discriminative performance of plasma NFL levels and clinical variables, either alone or in combination, for predicting conversion to MSA-C for individuals followed up for more than 3 years. (**a**) Models using each variable individually (RBD, oDz, and NFL). (**b**) Two-variable models combine each individual variable with age at sampling. (**c**) Two-variable models using combinations of clinical parameters. (**d**) Three-variable models, including age at sampling added to those in (**d**). (**e**) Models incorporating three or more variables. Age refers to age at the time of sample collection.

Abbreviations: AUC, area under the curve; MSA-C, Multiple system atrophy cerebellar type; oDz, orthostatic dizziness; NFL, neurofilament light chain; RBD, REM sleep behavior disorder; ROC, Receiver operating characteristic
